# On the puzzling case of sodium saccharinate 1.875-hydrate: structure description in (3+1)-dimensional superspace

**DOI:** 10.1107/S2052520619014938

**Published:** 2020-02-01

**Authors:** Toms Rekis, Andreas Schönleber, Sander van Smaalen

**Affiliations:** a Laboratory of Crystallography, University of Bayreuth, Universitätsstrasse 30, 95447 Bayreuth, Germany

**Keywords:** sodium saccharinate 1.875-hydrate, high-*Z*′ structures, superstructures, superspace, rigid-body modelling, commensurate structures

## Abstract

The structure of sodium saccharinate 1.875-hydrate is presented in three- and (3+1)-dimensional space. The present model is more accurate than previously published superstructures, due to an excellent data set collected up to a high resolution of 0.89 Å^−1^. The present study confirms the unusual complexity of the structure comprising a very large primitive unit cell with *Z*′ = 16. A much smaller degree of correlated disorder of parts of the unit cell is found than is present in the previously published models. As a result of pseudo-symmetry, the structure can be described in a higher-dimensional space. The X-ray diffraction data clearly indicate a (3+1)-dimensional periodic structure with stronger main reflections and weaker superstructure reflections. Furthermore, the structure is established as being commensurate. The structure description in superspace results in a four times smaller unit cell with an additional base centring of the lattice, resulting in an eightfold substructure (*Z*′ = 2) of the 3D superstructure. Therefore, such a superspace approach is desirable to work out this high-*Z*′ structure. The displacement and occupational modulation of the saccharinate anions have been studied, as well as their conformational variation along the fourth dimension.

## Abstract 2

A high-*Z*′ structure of sodium saccharinate 1.875-hydrate has been found to be a commensurately modulated structure well described in (3+1)-dimensional superspace.

## 1. Introduction

Usually, the lattice parameters, space-group symmetry and spatial arrangement of the atoms of the asymmetric unit are sufficient to describe a crystal structure considered as a static idealized state of matter. This formalism is the very essence of crystallography. For molecular crystals, in the vast majority of cases, there is only one molecule (or formula unit in the case of multi-component phases) in the asymmetric unit (*Z*′ = 1). It has been reported, however, that *Z*′ > 1 for around 8% of crystal structures (Steed, 2003 ▸). It is imaginable that sometimes larger aggregates can form from the constituent molecules. Such aggregates may possess no crystallographic symmetry, or if they do then it does not transfer to the space-group symmetry. For example, it does not seem unusual that efficiently interacting dimers or trimers could exist that are then packed according to some space-group symmetry. More intriguing, however, are cases for which *Z*′ is high (*i.e. Z*′ > 4; Brock, 2016 ▸). There have been a number of studies dealing with high-*Z*′ structures (Steed, 2003 ▸; Steed & Steed, 2015 ▸; Desiraju, 2007 ▸; Brock, 2016 ▸) and one of the conclusions is that sometimes such structures must be described as modulated, as considerable pseudo-symmetry can be identified.

Since the pioneering work of de Wolff and co-workers (Brouns *et al.*, 1964 ▸; de Wolff, 1974 ▸) and Janner & Janssen (1977 ▸), modulated structures are well understood and the mathematical apparatus has been well established (Janssen *et al.*, 2007 ▸; van Smaalen, 2007 ▸; Petricek *et al.*, 1985 ▸). However, they perhaps do not gain the attention they deserve, especially in the field of chemical crystallography where the studied systems constitute molecular compounds (Schönleber, 2011 ▸). Moreover, high-*Z*′ structures of molecular crystals, should they give any indication of the presence of possible modulation, must be treated very cautiously. For example, incommensurately modulated structures may sometimes be relatively well approximated by a sufficiently large supercell in the conventional 3D space (Schönleber & Chapuis, 2004 ▸; Wagner & Schönleber, 2009 ▸). However, such an approximated structure model is fundamentally wrong. A description of a commensurately modulated structure in 3D space is fully valid, but a more sophisticated approach in a higher-dimensional space is found to be much more suitable when, for example, consecutive phase transformations including low-*Z*′ phases are to be analysed (Zuñiga *et al.*, 1989 ▸; Harris *et al.*, 1994 ▸; Schönleber *et al.*, 2003 ▸) or the origins of the structure formation are to be explained (Dey *et al.*, 2016 ▸, 2018 ▸).

**Figure 1:**
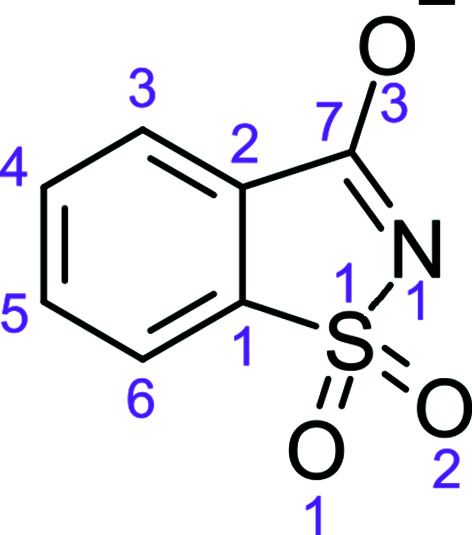
The chemical scheme of the saccharinate anion, with the atom numbering used in this study.

One example of a molecular crystal structure with a high *Z*′ value is that of sodium saccharinate 1.875-hydrate. The saccharinate chemical scheme is presented in Fig. 1 ▸. In spite of this artificial sweetener having been used in the food industry for over 100 years (Baran & Yilmaz, 2006 ▸), its crystal structure has been published only relatively recently (Naumov *et al.*, 2005 ▸; Banerjee *et al.*, 2005 ▸). The reason for that might be the unusual complexity of this *Z*′ = 16 structure. It is surprising that the large number of crystal constituents, comprising 16 saccharinate anions, 16 sodium cations and 30 water mol­ecules, could be consistently arranged in a reproducible manner, replicated according to the space-group symmetry and then translated in all three directions. Furthermore, some of the entities in the asymmetric unit are disordered. The two independently elucidated structure models for crystals measured at around the same temperature (95 and 100 K) and published at nearly the same time are virtually identical, including the occupancies of the disordered sites. This indicates that the particular structural complexity of this organic salt hydrate is prevalent and does not arise from some specific experimental conditions of crystal growth or other non-equilibrium processes. In one of the studies (Naumov *et al.*, 2005 ▸) it was pointed out that some pseudo-symmetry is present, but this only fully applies to the saccharinate ions. Nevertheless, it means that the structure could be described in a higher-dimensional space with a smaller unit cell and thus higher symmetry.

The aim of this study is to gain a deeper understanding of the sodium saccharinate 1.875-hydrate structure by describing it within the superspace approach and establishing its true commensurate or incommensurate nature.

## 2. Experimental

### 2.1. Materials

Sodium saccharinate hydrate (assay ≥99%) was purchased from Sigma–Aldrich and used without further purification. Deionized water was treated in a Millipore purification system. Single crystals were grown by slow evaporation from water solution. Transparent prismatic crystals of different sizes were obtained.

### 2.2. X-ray diffraction data collection

Single-crystal X-ray diffraction data were collected on a mar345dtb diffractometer equipped with an image-plate detector. A Bruker–Nonius rotating-anode X-ray generator was used with a wavelength of 0.56089 Å (Ag *K*α radiation). A crystal of dimensions 0.22 × 0.35 × 0.45 mm was glued onto a glass fibre and coated with a thin layer of glue to prevent dehydration of the sample. An open-flow nitrogen cryostat was exploited for temperature regulation and the sample was cooled to 95 K with a cooling rate of 2 K min^−1^. φ scans were performed with a step size of 1°. In total 360 frames were collected. An exposure time of 100 s per frame was used.

Data indexing and integration (with respect to the supercell structure previously reported in *P*2_1_/*n*; Naumov *et al.*, 2005 ▸; Banerjee *et al.*, 2005 ▸) was performed up to the diffraction limit of 0.89 Å^−1^ with the *CrysAlisPro 39.46* software (Rigaku Oxford Diffraction, 2018 ▸). Outlier rejection according to the Laue class 2/*m* was applied. Empirical absorption correction using spherical harmonics, implemented in the *SCALE3 ABSPACK* scaling algorithm within the *CrysAlisPro 39.46* software, was applied. The data are listed in Table 1 ▸.

### 2.3. Structure refinement

#### 2.3.1. Supercell structure

**Figure 2:**
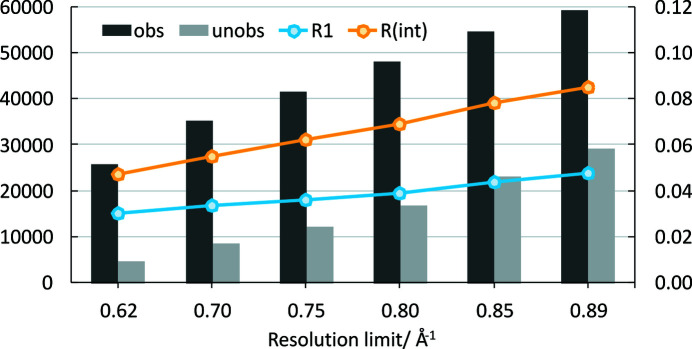
The number of observed and unobserved reflections [according to *I* > 3σ(*I*)], *R*_1_ and *R*_int_ (all cumulative) versus the resolution limit [sin(θ)/λ]_max_ used for refinement.

Structure refinements were performed with the software *JANA2006* (Petříček *et al.*, 2014 ▸). Initial non-H atom positions were taken from a published structure model (Banerjee *et al.*, 2005 ▸). Non-H atoms were refined anisotropically. H atoms were added geometrically or located from the difference Fourier maps. Riding isotropic atomic displacement parameters (ADPs) were applied. No H atoms could be located for a few disordered water molecules with reduced site-occupancy factors. The data are listed in Table 2 ▸. The low *R*_1_ and *R*_int_ values indicate the excellent fit of the refined structure model to the diffraction data. Furthermore, the use of silver radiation allowed the collection of good data up to an exceptionally high resolution of 0.89 Å^−1^ for this light-atom structure. This is particularly important for an appropriate refinement of *e.g.* the ADPs. In Fig. 2 ▸ the number of observed and unobserved reflections [according to *I* > 3σ(*I*)] and the *R*_1_ and *R*_int_ values (all cumulative) are plotted versus the resolution limit [sin(θ)/λ]_max_. The *R*_1_ value drops down to around 0.03 if the conventional limit for light-atom structures of 0.62 Å^−1^ is chosen. At this limit, the *R*_1_ values obtained in previous studies are 0.08 (Naumov *et al.*, 2005 ▸) and 0.045 (Banerjee *et al.*, 2005 ▸).

**Figure 3:**
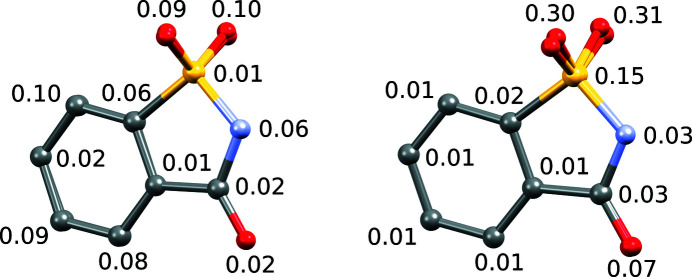
Best overlays (left: all atoms are matched; right: only phenyl ring atoms are matched) of molecules 1 and 11, showing their non-identical conformations. Deviations between corresponding atoms are given in Å.

Owing to the excellent data quality, subtle differences in saccharinate anion conformations were noted. Although it is an aromatic and therefore seemingly rigid molecule, the forces of the different crystallographic environments deform the 16 independent molecules significantly. Using the rigid-body approach for the whole molecule, the *R*_1_ value doubled in magnitude. Although this approach is very appealing, since it reduces the number of refinement parameters from 2387 to 1105 or even lower (Naumov *et al.*, 2005 ▸), if the probable mirror plane through the molecule is considered then the conformational variance of the saccharinate anions throughout the crystal structure cannot be neglected. The rigidity of the molecule was studied by generating best overlays of every two molecules. It was found that there can be a deviation as large as 0.1 Å between corresponding atoms (see Fig. 3 ▸, where the best overlay of molecules 1 and 11 is depicted). Such deviations can be considered large and are most certainly reflected in the increase in the *R*_1_ value when the rigid-body approach is applied for the molecule as whole.

It was, however, found that the phenyl ring can be considered rigid. The best overlays of the phenyl ring atoms (taken from the refined structure with no restrictions applied to the atomic positions) showed that the corresponding atoms are usually not more than around 0.01 Å apart (see Fig. 3 ▸), and this is also confirmed by calculating the best planes through the six atoms. The distances of the saccharinate anion atoms from the best planes through the corresponding phenyl rings are listed in Table S1 in the supporting information.

In the final structure model a rigid-body approach was applied to the phenyl ring, defining it as a set of six coplanar atoms, but refining its internal structure in terms of bond lengths and angles. The *R*_1_ value increased only slightly compared with that of the free-atom model, confirming that the geometries of the phenyl rings are consistently identical and are not influenced by the different crystallographic environments. The number of refinement parameters could be decreased from 2394 to 2016.

Furthermore, one saccharinate anion, three sodium cations and four water molecules are each disordered over two sites. In the previously reported structure models the occupancies were restricted to be equal for all atoms of the major site and all atoms of the minor site with a sum of unity. The reported occupancy ratios from those studies are 0.67:0.33 (Naumov *et al.*, 2005 ▸) and 0.70:0.30 (Banerjee *et al.*, 2005 ▸). It was also established in the present study that the disorder is correlated, as refining it separately (only restricting the sum to be unity for a major and the corresponding minor site) revealed that it is very similar among all the major (and minor) components. The refined unified ratio of 0.88:0.12 for the correlated disordered entity is, however, different from that reported previously.

#### 2.3.2. Superspace structure

From the established superstructure model of sodium saccharinate 1.875-hydrate it was noted that there is pseudo-translation symmetry present in the *P*2_1_/*n* unit cell. The particular crystal structure can be described in (3+1)D superspace with the basic cell (bc) and the present supercell (sc) being related by the following transformation:

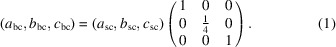

The superspace structure is an eightfold substructure of the one described in 3D space, as the former is represented by a four-times smaller unit cell with an additional base-centring of the lattice.

**Figure 4:**
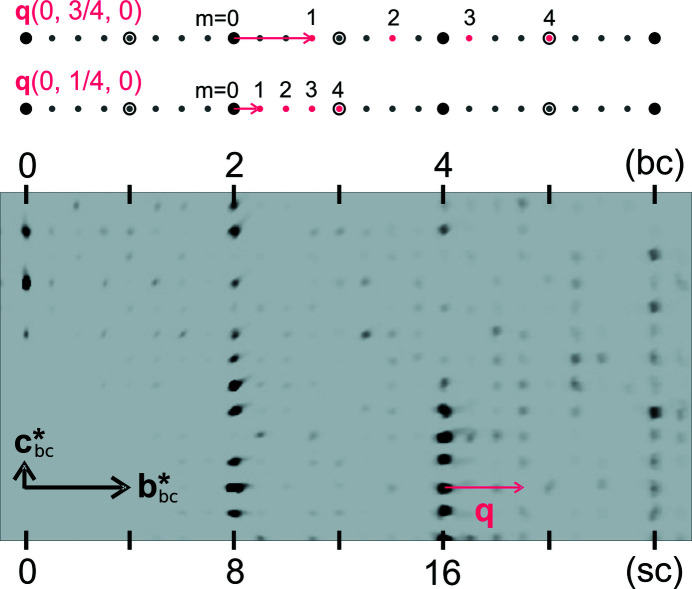
Section (0*kl*) of the reciprocal-space reconstruction of the experimental data, with *k* indices according to the basic cell (bc) and supercell (sc). The top panel gives a schematic representation of possible indexing, alternately based on different **q** vectors. Large circles indicate main reflections, small circles satellite reflections, open circles absent reflections and full circles allowed reflections.

In Fig. 4 ▸ a section of the reciprocal layer 0*kl* is shown, as well as a schematic representation of possible satellite reflection indexing. The 0*kl* section features strong main reflections and systematically absent reflections according to the *C*-centre condition *h* + *k* = 2*n*. The modulation wavevector **q** lies along the b* axis. Assuming that the structure is truly commensurate, the value of **q** is either ( 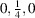 ) or ( 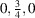 ). Thus, up to the fourth order satellites are present, with *m* = ±4 satellites coinciding at the positions of the absent main reflections. It can be noted in Fig. 4 ▸ that the satellite reflections at ±¾ distance from the present main reflections appear stronger than those at ±¼ distance, which are barely visible. Inspection of the intensity statistics of the complete reflection list reveals that the modulation wavevector is most favourably defined as ( 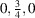 ) (see Table 3 ▸). The presence of the *C*-centre is also evident. The intensity statistics and observations in Fig. 4 ▸ are an indication that this structure is well described in superspace.

**Figure 5:**
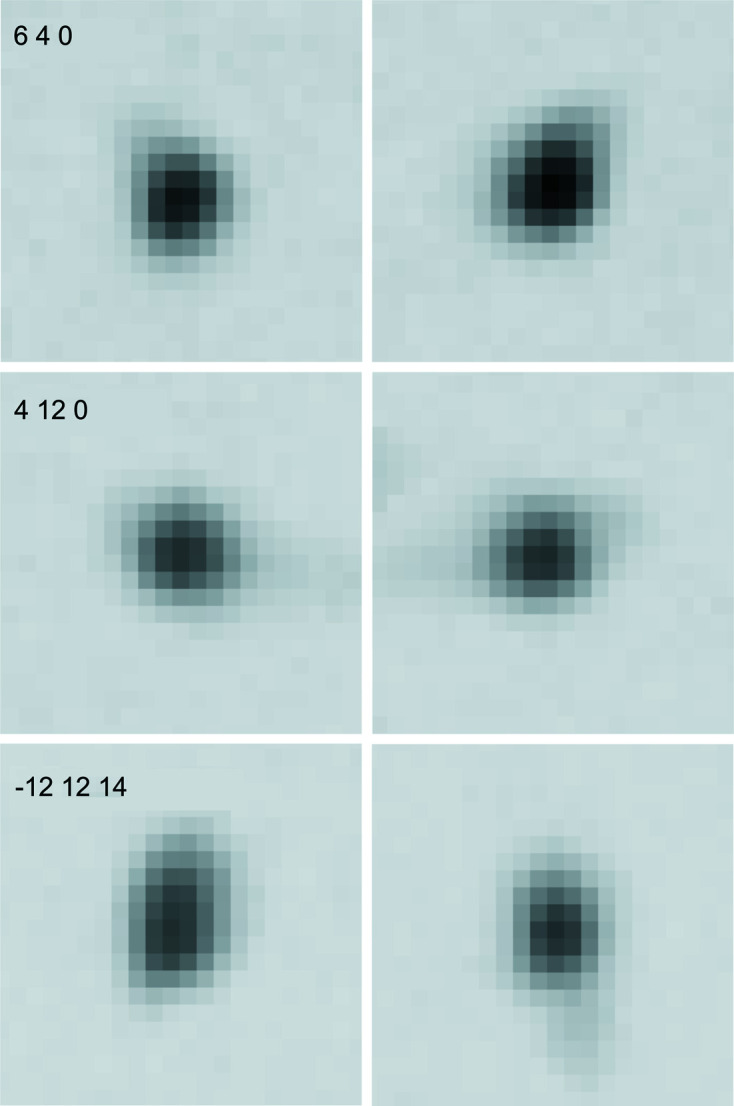
Pairs of equivalent strongest fourth-order satellite reflections ( 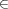 4*h* + *k* = 8*n* + 4, indices with respect to the supercell). Slight splitting of the 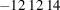 reflections is associated with the combination of non-monochromaticity of the incident beam and the geometry of the data collection at higher 2θ angles.

Although there is a proper solution of the structure in 3D space suggesting commensurability, it cannot be excluded that the structure might be incommensurate. One indication for that would be splitting of the fourth-order satellite reflections, if the component σ_2_ of the modulation wavevector **q** were to deviate from the rational number of ¾. Otherwise (*h*, *k*, *l*, 4) and (*h*, *k* + 6, *l*, −4) describe the same point in reciprocal space (see Fig. 4 ▸). Careful examination of the strongest fourth-order satellite reflections in the diffraction data frames showed single peaks with good shapes. Minor splitting of a few peaks recorded at higher 2θ angles was associated with non-monochromaticity of the incident beam. In Fig. 5 ▸ a few strongest fourth-order satellite reflections located in the raw data frames are shown, confirming the above statement. Commensurability of the structure at the analysed temperature was therefore established, and ultimately proven upon structure refinement.

Because of this commensurability, the reflection data for structure refinement in (3+1)D space could be re-indexed by an in-house script using the reflection list from the supercell structure refinement, therefore avoiding new data integration, scaling and corrections. In such a way, different refinement parameters can be more straightforwardly related to those of the refinement in 3D space.

A space-group symmetry test in the *JANA2006* software suite indicated the superspace group *C*2/*c*(0,σ_2_,0)*s*0 [standard setting *B*2/*b*(0,0,σ_3_)*s*0, No. 15.1.7.3 (Stokes *et al.*, 2011 ▸; van Smaalen *et al.*, 2013 ▸)]. An origin shift of (¼, ¼, 0, 0) was applied so that the fractional coordinates of the atoms correspond to those of the superstructure. The resulting symmetry operators are: *x*_1_, *x*_2_, *x*_3_, *x*_4_; −*x*_1_, *x*_2_ + ½, −*x*_3_ + ½, *x*_4_ + ½; −*x*_1_, −*x*_2_, −*x*_3_, −*x*_4_; *x*_1_, −*x*_2_ + ½, *x*_3_ + ½, −*x*_4_ + ½. Regarding commensurability, there are three inequivalent options for *t*_0_, namely general, *t*_0_ = 0 and *t*_0_ = 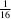 . These correspond to the supercell space groups *P*2_1_, *P*2_1_/*n* and *P*2_1_/*c*, respectively. The value *t*_0_ = 0 was selected, as the presented unit cell is indexed in such a way as to correspond to *P*2_1_/*n*.

Structure solution with *SUPERFLIP* (Palatinus & Chapuis, 2007 ▸) gave a satisfying starting model. However, such *ab initio* refinement did not lead to an accurate structure model. Apparently, this failure is due to the presence of an intricate occupational modulation in a crenel function fashion. Initial coordinates for the basic positions and parameters for the modulation and crenel functions were then derived from the supercell structure model. Average positions were used as initial basic positions and, for most atoms, up to fourth-order harmonics were necessary to describe their positional modulation properly. Each harmonic adds up to 2 × 3 parameters, meaning that (together with the three basic position coordinates) three independent atomic positions of the supercell can be described. Coefficients for the highest-order harmonic sine or cosine terms were fixed to 0 values when necessary so as not to exceed the required number of parameters and thus avoid singularities, for example for atoms describing eight independent positions in the supercell. In such a way the initial structure model in (3+1)D superspace was set up for the refinement.

Because of the eightfold relation between the cells and *Z*′ of 16 for the supercell, the basic cell comprised two saccharinate anions (atoms C1*A*/*B*, C2*A*/*B*, C3*A*/*B*, C4*A*/*B*, C5*A*/*B*, C6*A*/*B*, C7*A*/*B*, S1*A*/*B*, N1*A*/*B*, O1*A*/*B*, O2*A*/*B*, O3*A*/*B*), four sodium cation sites (Na2, Na6, Na14, Na16) and four water O atom sites (O18*W*, O23*W*, O24*W*, O28*W*) in the asymmetric unit. The occupancy of the sodium cations along the *x*_4_ coordinate is thus zero for some intervals as, according to the stoichiometry, formally only two are required for the two saccharinate anions. The situation is similar for the O atom O23*W* as the hydrate stoichiometry is not exactly two, requiring fewer than four water molecules per two saccharinate anions.

Several additional atoms were included with respective crenel functions to account for the disorder. As for the 3D model, a rigid-body approach was used for the phenyl rings of the two saccharinate ions. Non-H atoms were refined anisotropically applying modulated ADPs using the same order as the corresponding order for positional modulation. Hydrogen atoms of the saccharinate ions were added geometrically. TLS parameters were used for the phenyl ring carbon atoms and their respective H atoms. The ADP values of the minor disorder component of the saccharinate anion were constrained to be equal to those of the major component. No H atoms of the water molecules were added. Crystallographic and refinement data are listed in Table 2 ▸. The larger *R*_1_ value compared with the 3D model is explained by the absence of water H atoms.

### 2.4. Density functional theory (DFT) calculations

The single-point energy of the saccharinate anions was calculated with the program *GAUSSIAN09* (Frisch *et al.*, 2009 ▸). Atomic coordinates were taken from the superstructure refinement with no restraints. H-atom distances were renorm­alized to 1.09 Å. The M062X level of theory and 6-311++g(d,p) basis set were used. No calculations were performed for the two saccharinate anions superimposed at the disordered site, since the atomic positions are not accurate enough. An additional calculation was performed allowing the atomic positions to relax to obtain the gas-phase reference energy. The obtained energy values are listed in Table S2.

## 3. Results and discussion

### 3.1. Superstructure of sodium saccharinate 1.875-hydrate

**Figure 6:**
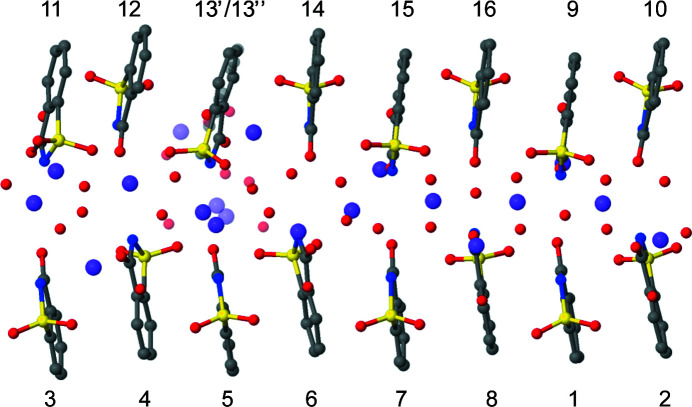
The asymmetric unit of sodium saccharinate 1.875-hydrate at 95 K, showing the molecule numbering scheme. Minor components of the disordered sites are depicted as partly transparent. C atoms (gray), O atoms (red), N atoms (blue), S atoms (yellow), Na ions (violet).

The asymmetric unit of the title compound at 95 K described in space group *P*2_1_/*n* is depicted in Fig. 6 ▸. There are a total of 16 saccharinate anions, 16 sodium cations and exactly 30 water molecules. The arrangement of the ions and mol­ecules in the asymmetric unit is somewhat puzzling, as only a part of it can be considered modulated. The saccharinate anions are consistently stacked in a column along **b**. A regular arrangement is also present for approximately half of the sodium cations and water molecules. However, there is a strong deviation from this modulated pattern as the remaining sodium cations and water molecules are positioned very differently. Furthermore, there is disorder present concerning molecule 13 and neighbouring sodium cations and water molecules. This rather irregular pattern indicates that it is a somewhat of a compromise solution for the crystal structure to form.

It is intriguing that such a large, complex and not even fully pseudo-symmetrically consistent aggregate can apparently be found as a regular pattern throughout the crystal structure. The supramolecular arrangement, including the sophisticated disorder of one saccharinate anion, three sodium cations and four water molecules, is identical for several crystals tested within this study. Moreover, it is virtually the same as that found in the previous studies of this very same solid phase at 95 and 100 K (Naumov *et al.*, 2005 ▸; Banerjee *et al.*, 2005 ▸). This rules out the possibility that this high-*Z*′ structure would be any kind of fossil relic of the solution aggregation process as proposed for some high-*Z*′ compounds (Steed, 2003 ▸; Desiraju, 2007 ▸). The most notable difference between the present model and previous models is the disorder, with an occupancy ratio of 0.88:0.12 compared with that of around 0.70:0.30 reported previously (Naumov *et al.*, 2005 ▸; Banerjee *et al.*, 2005 ▸).

### 3.2. Superspace structure of sodium saccharinate 1.875-hydrate

#### 3.2.1. Modulation of the asymmetric unit entities

Due to the eightfold relation between the unit cells in 3D and (3+1)D space (see the *Experimental* section[Sec sec2]), the asymmetric unit of the superspace structure (*Z*′ = 2) formally consists of two saccharinate anions, two sodium cations and 3.75 water molecules. However, the basic structure is more complex than that.

**Figure 7:**
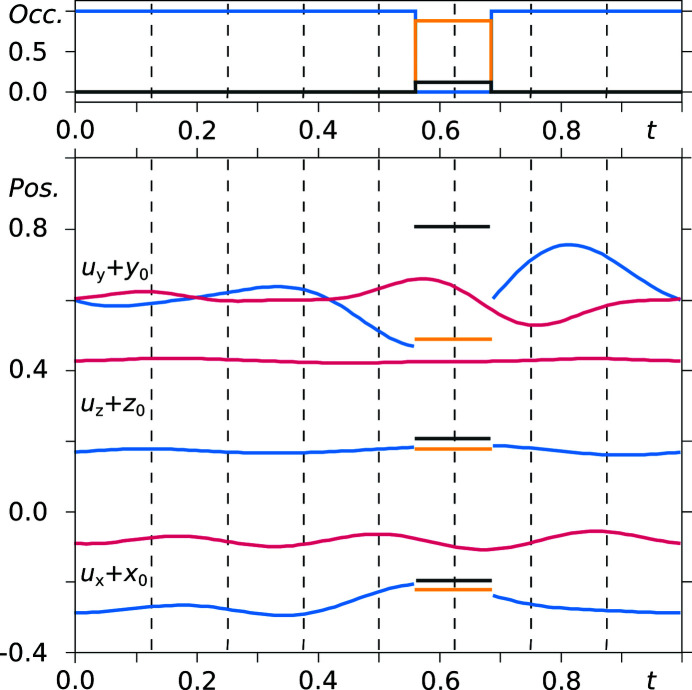
*t*-Plots of the positional (bottom) and occupational (top) modulation of atoms O1*A* (vermillion), O1*B* (blue), O1*B*′ (yellow), O1*B*′′ (grey). Commensurate *t*-sections are indicated by dashed lines.

There are indeed two saccharinate anions defined, which are denoted *A* and *B* (see Fig. 1 ▸ for the atom numbering). Modulation functions are applied for entity *A* to define the eight saccharinate positions along the *x*_4_ direction, and similarly for entity *B*. However, at a certain *t* value the anion is disordered over two positions, hence additional basic positions of the saccharinate anion with reduced site occupancy have been defined, namely *B*′ and *B*′′. Corresponding crenel functions to alter the *B*/*B*′/*B*′′ occupancy between 0 and the respective non-zero value have been defined, and these are summarized in a *t*-plot of the modulations of atoms O1*A* and O1*B* as an example (Fig. 7 ▸). The largest modulation amplitudes are found for modulation functions *u_y_*, but the corresponding unit-cell dimension *b* is several times smaller than *a* and *c* (see Table 2 ▸). Modulation functions *u_z_* are approximately constant throughout the modulation period. For the saccharinate anion *B* the dis­ordered molecular site occurs at *t* = 0.625 where the saccharin­ate anion adopts two slightly different orientations with an occupancy ratio of 0.88:0.12 (corresponding to saccharinate 13 in the superstructure). Only a single position needs to be described for *B*′ and *B*′′, which is already defined by the corresponding basic positions (*x*, *y* and *z* values), so no additional displacive modulation function need be added for the atoms of the *B*′ and *B*′′ entities.

It can be noted, however, that the position of atom O1*B*′ fits relatively well within the modulation curve of O1*B*. Restricting the *B*′ atoms to have the same basic positions and modulation functions as the *B* atoms was found to be not feasible since such a refinement resulted in unreasonable basic positions for some atoms and an increase in *R*_1_. *t*-Plots for atoms O1*B*, O2*B*, O3*B*, S1*B*, N1*B* and C7*B* defining the flexible part of the molecule (and their *B*′ counterparts) are given in the supporting information, showing that the *B*′ positions deviate rather significantly from the assumed positions according to the modulation function of the *B* atoms.

It must be added that for the other saccharinate atoms the modulation functions are very similar to each other and possess the same characteristics, as shown for atoms O1*A* and O1*B* in Fig. 7 ▸.

**Figure 8:**
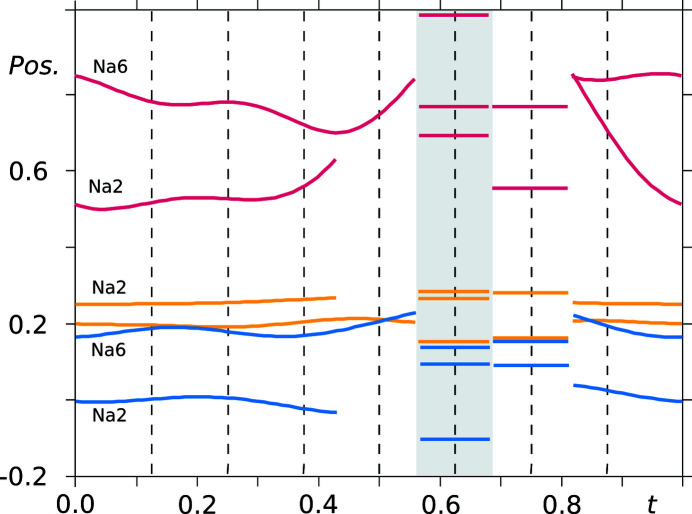
*t*-Plots of the positional modulation of sodium cations (vermillion: *u_y_* + *y*_0_; blue: *u_x_* + *x*_0_; yellow: coordinate *u_z_* + *z*_0_). Atoms in the shaded area are disordered over two sites and only the major occupancy site is depicted. Commensurate *t*-sections are indicated by dashed lines.

Regarding the sodium cations in the asymmetric unit, the picture is more complex (see Fig. 8 ▸). For the two asymmetric sodium ions named Na2 and Na6 there is a severe discontinuity in their modulation functions. Additional sodium cations have therefore been defined in limited crenel intervals. Furthermore, there is an inconsistency in the ion density in the *t*-sections, as apparently one of the sodium ions is absent at section *t* = 0.5 and instead an additional cation appears at *t* = 0.625. Finally, all three atoms at this particular *t*-section are disordered over two sites.

**Figure 9:**
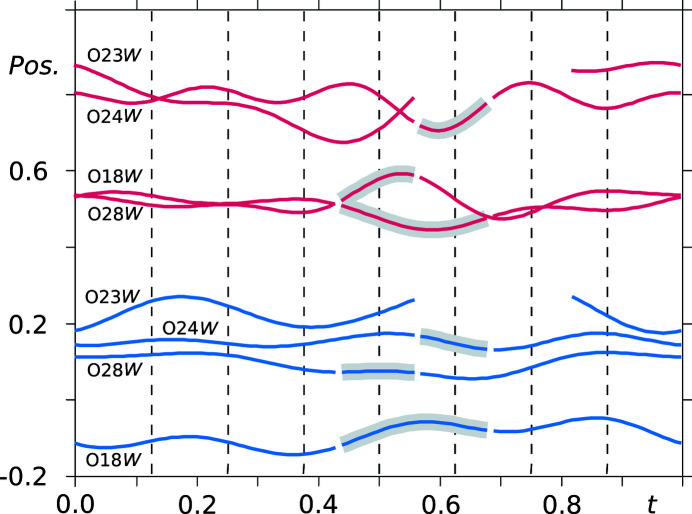
*t*-Plots of the positional modulation of water molecule O atoms (vermillion: *u_y_* + *y*_0_; blue: *u_x_* + *x*_0_). Atoms in the shaded areas are disordered over two sites and only the major occupancy site is depicted. Commensurate *t*-sections are indicated by dashed lines.

The modulation of the water molecules (or their respective O atoms) is depicted in Fig. 9 ▸. The component *u_z_* has been omitted for the sake of clarity. Furthermore, *u_z_* is approximately constant, similar to the previously discussed atoms. For one of the water molecules there are two sites empty from the eight sites within one period along the *x*_4_ axis, resulting in the unusual stoichiometry of 1:1.875 between the saccharinate (sodium) ions and water molecules. At four *t* values (shaded grey) the water molecules are disordered over two sites. Here, the major occupancy sites have been restrained to have the same basic positions and modulation function parameters as atoms O18*W*, O28*W* and O24*W*, respectively.

From the *t*-plots discussed above it has been shown that half of the sodium ions, in particular, are inconsistently positioned throughout the structure, albeit in a periodic fashion. The rather odd cation placement and disorder around the section *t* = 0.625 correlates with the absence and disorder of the water molecules around the same section. Regarding the independent saccharinate anions, the inconsistencies are not so severe, except for the presence of a disordered site and larger deviations from the basic positions in terms of *y* coordinate (see Fig. 7 ▸).

#### 3.2.2. Variation in the saccharinate anion conformation

**Figure 10:**
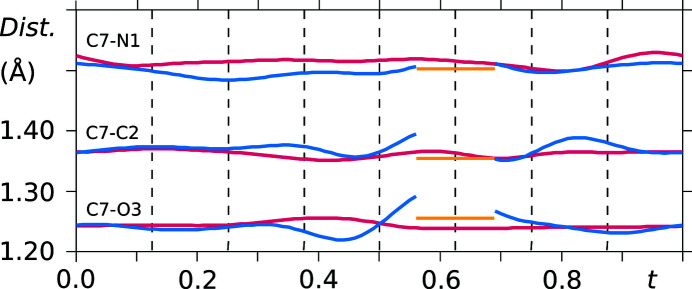
*t*-Plots of the interatomic distances between atom C7 and the respective covalently bonded atoms N1, O3 and C2 (vermillion: molecule *A*, blue: molecule *B*, yellow: major component of the disordered site). Commensurate *t*-sections are indicated by dashed lines.

**Figure 12:**
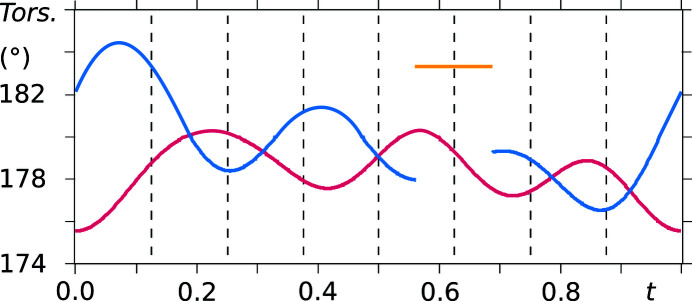
*t*-Plots of the torsion angle S1—N1—C7—O3 (vermillion: molecule *A*, blue: molecule *B*, yellow: major component of the disordered site). Commensurate *t*-sections are indicated by dashed lines.

As already noted in the analysis of the supercell model, the saccharinate anion geometry varies throughout the crystal structure, except for the phenyl ring. The conformational variation of the two independent saccharinate anions can be noted in *t*-plots where the bond lengths between the constituent atoms are depicted (see Figs. 10 ▸ and 11 ▸). Furthermore, the molecule is not planar, and therefore no internal mirror symmetry can be exploited to reduce the number of refinement parameters. This is proven by *t*-plots showing the torsion angles S1—N1—C7—O3 and N1—C7—O3—phenyl (Figs. 12 ▸ and 13 ▸). Torsion-angle values for S1—N1—C7—O3 span ranges of around 176°–180° and 176°–184° for molecules *A* and *B*, respectively, indicating non-planarity of the saccharin­ate heterocycle. Furthermore, the planar phenyl ring is situated at angles in the ranges of 2°–4° and 2°–6° with respect to the plane N1—C7—O3 for molecules *A* and *B*, respectively.

**Figure 14:**
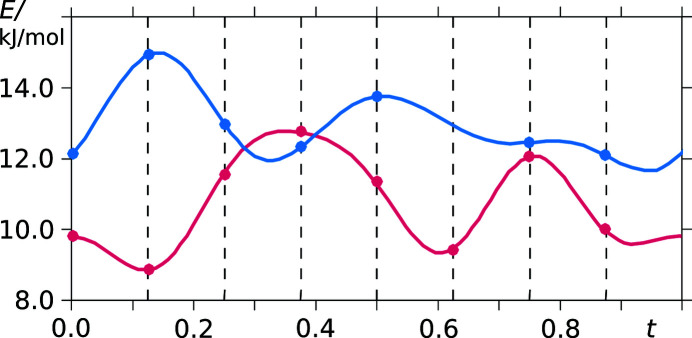
Conformer energy versus *t* (vermillion: molecule *A*, blue: molecule *B*). Fourth-order harmonics were used to fit the data points.

DFT calculations of conformer energies show that they vary between 9 and 15 kJ mol^−1^ compared with the gas-phase conformation (see Table S2). The energy variance for both asymmetric saccharinate anions *A* and *B* versus the modulation period *t* is depicted in Fig. 14 ▸. The energy of the site-*A* saccharinate anions is consistently lower than that of the *B* anions. This indicates that there is a smaller frustration exerted on the site-*A* molecules. However, the difference in conformer energy of at least 9 kJ mol^−1^ compared with the gas-phase conformation shows that the crystal environment exerts a considerable force on the saccharinate anions.

#### 3.2.3. Sodium coordination spheres

**Figure 15:**
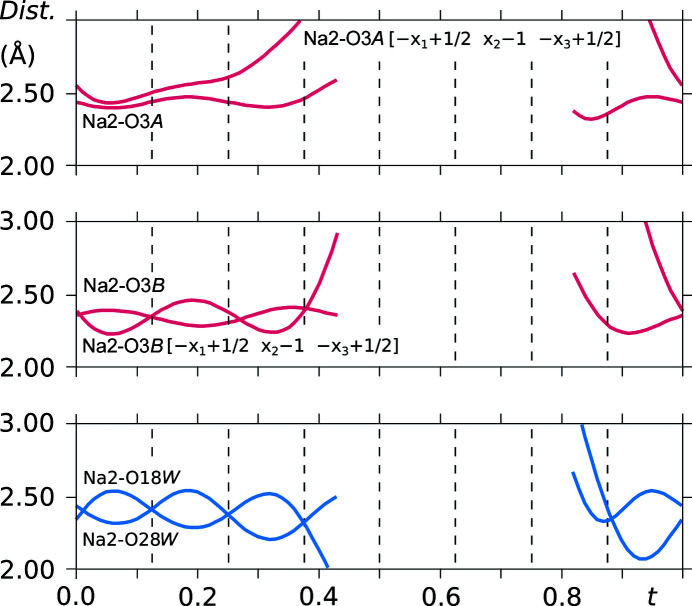
*t*-Plots of the interatomic distances in the Na2 coordination sphere. Commensurate *t*-sections are indicated by dashed lines.

Most of the sodium ions are octahedrally coordinated. Sodium cation Na2 is coordinated by water molecules O18*W* and O28*W* and by saccharinate anions through carbonyl O atoms O3*A* and O3*B*. Interatomic distances are presented in Fig. 15 ▸. Opposite water molecules O18*W* and O28*W* are more consistently located equidistant from the Na2 cation. Deviations from an optimal distance to the central atom are more pronounced in the case of the O atoms of the saccharinate ion, especially those of the asymmetric molecule *B*.

**Figure 16:**
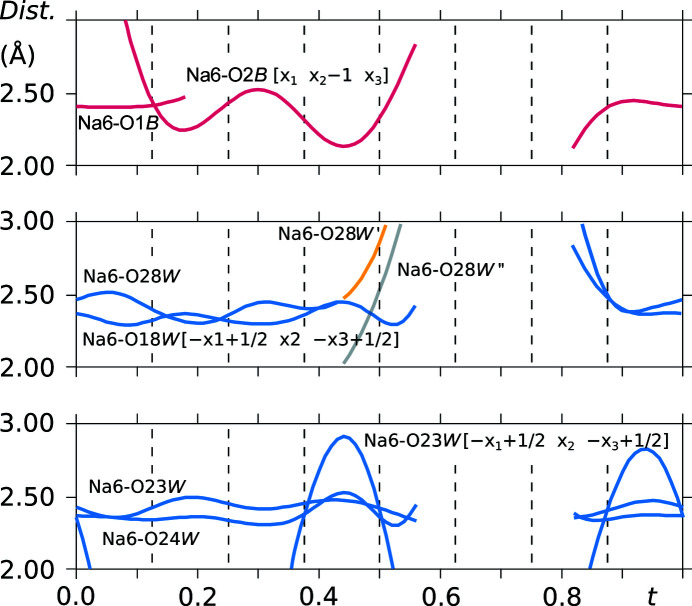
*t*-Plots of the interatomic distances in the Na6 coordination sphere. Commensurate *t*-sections are indicated by dashed lines.

Sodium cation Na6 is coordinated by all four asymmetric water molecules, namely, O18*W*, O23*W*, O24*W* and O28*W*. It also interacts with some of the sulfonyl group O atoms of saccharinate anion *B* (Fig. 16 ▸).

The remaining sodium cations Na2′, Na14, Na14′, Na16 and Na16′, and the respective minor sites Na14′′ and Na16′′, all interact with neighbouring water molecules and with the O and N atoms of the saccharinate anions, but do not show clear octahedral coordination polyhedra.

## 4. Conclusions

The analysis of X-ray diffraction data intensity statistics shows that the structure of sodium saccharinate 1.875-hydrate is well described in (3+1)D superspace as a commensurate case in a *C*-centred lattice with a modulation wavevector **q** of (0, 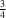 , 0). The centred unit cell in the superspace group *C*2/*c*(0, σ_2_, 0)*s*0 corresponds to an eightfold substructure of a four times larger primitive 3D supercell in *P*2_1_/*n*. The superspace approach allows *Z*′ to be reduced from 16 to 2, while the 2 × 8 = 16 different conformations are described by modulation functions. The 3D model is in accordance with the two previously published models, but it is more accurate due to better data quality and considerably higher resolution.

The structure of the title compound possess an intricate organization of constituent entities and it is accompanied by a correlated disorder. Although the saccharinate anion is aromatic and seemingly rigid, a subtle conformation variance has been found involving the heterocyclic part of the molecule. The organic anions are organized uniformly throughout the crystal structure. The sodium cations, by contrast, are only partly consistently positioned. This can be explained by the fact that such single-atom ions can equally well interact with counter-ions in any direction, whilst for the saccharinate anions there is steric hindrance present, and only a limited volume in space around the negatively charged amide entity is accessible to form a favourable interaction. The water mol­ecules follow the arrangement of the sodium ions as they are coordinated around them. The unusual hydrate stoichiometry, however, indicates that they are rather acting as void fillers. It is not clear what causes around half of the 16 crystallographically independent sodium ions not to obey the pseudo-symmetrical arrangement as found for the rest. The overall structure is rather surprising, as such a complex and large asymmetric arrangement consistently spans across the crystal structure.

## Supplementary Material

Crystal structure: contains datablock(s) global, I, II. DOI: 10.1107/S2052520619014938/dk5088sup1.cif

Structure factors: contains datablock(s) I. DOI: 10.1107/S2052520619014938/dk5088Isup2.hkl

Structure factors: contains datablock(s) II. DOI: 10.1107/S2052520619014938/dk5088IIsup3.hkl

Supporting information file. DOI: 10.1107/S2052520619014938/dk5088sup4.pdf

CCDC references: 1963817, 1963818

## Figures and Tables

**Figure 11:**
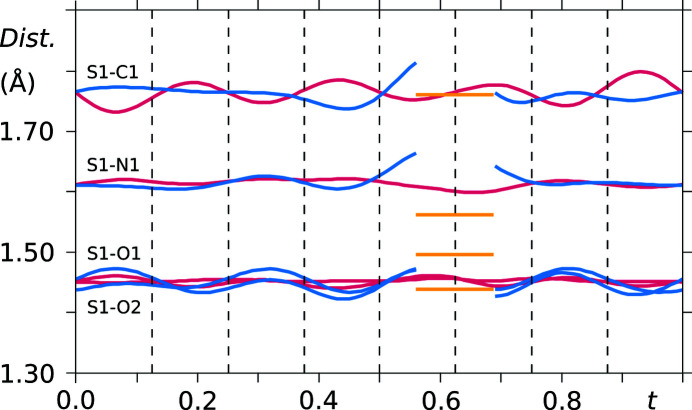
*t*-Plots of the interatomic distances between atom S1 and the respective covalently bonded atoms C1, O1, O2 and N1 (vermillion: molecule *A*, blue: molecule *B*, yellow: major component of the disordered site). Commensurate *t*-sections are indicated by dashed lines.

**Figure 13:**
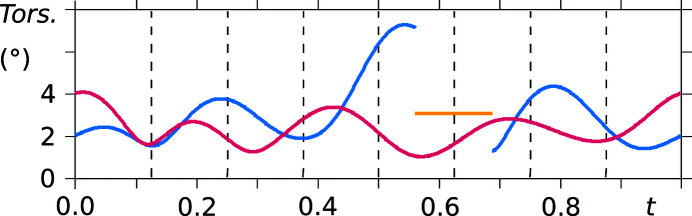
*t*-Plots of the torsion angle N1—C7—O3—phenyl (vermillion: molecule *A*, blue: molecule *B*, yellow: major component of the disordered site). Commensurate *t*-sections are indicated by dashed lines.

**Table 1:** Summary of the X-ray diffraction data acquisition

Temperature (K)	95
Radiation type, wavelength (Å)	Ag *K*α, λ = 0.56089
No. of reflections (cell measurement)	6894
θ range (°) (cell measurement)	2.84–29.89
Crystal size (mm)	0.22 × 0.35 × 0.45
*T*_min_, *T*_max_	0.912, 1
No. of measured, unique and observed [*I* > 3σ(*I*)] reflections	651 280, 88 532, 59 317
*R*_int_ (observed), *R*_int_ (unique)	0.0691, 0.0856
θ range (°) (data collection)	2.84–29.89
[sin(θ)/λ]_max_ (Å^−1^)	0.89
Index ranges	−32 → *h* → 32
	−50 → *k* → 50
	−51 → *l* → 51

**Table 2:** Crystallographic data for the structure models in 3D and (3+1)D space

Parameter	3D	(3+1)D
Crystal data		
Formula	Na(C_7_H_4_NO_3_S)·1.875H_2_O	
Formula weight	238.94	
Temperature (K)	95	
Crystal system	Monoclinic	
Space group	*P*2_1_/*n*	*C*2/*c*(0,σ_2_,0)*s*0
**q**, *t*_0_		(0, 3/4, 0), 0
*a* (Å)	18.62120 (10)	18.62120 (10)
*b* (Å)	28.4622 (2)	7.11555 (5)
*c* (Å)	29.1642 (2)	29.1642 (2)
β (°)	93.4511 (6)	93.4511 (6)
*V* (Å^3^)	15429.00 (17)	3857.25 (4)
*Z*, *Z*′	64, 16	16, 2
*F*(000)	7845	1904
*D_x_* (g cm^−3^)	1.6446	1.6446
μ (mm^−1^)	0.201	0.201
		
Refinement		
Refinement method	Full-matrix least-squares on *F*	
*R*_1_ [*F* ^2^ > 3σ(*F* ^2^)], *wR*(*F* ^2^), *S*	0.048, 0.055, 1.57	0.054, 0.065, 1.87
*R*_*m*=0_ [*F* ^2^ > 3σ(*F* ^2^)]		0.050
*R*_*m*=±1_ [*F* ^2^ > 3σ(*F* ^2^)]		0.052
*R*_*m*=±2_ [*F* ^2^ > 3σ(*F* ^2^)]		0.055
*R*_*m*=±3_ [*F* ^2^ > 3σ(*F* ^2^)]		0.056
*R*_*m*=±4_ [*F* ^2^ > 3σ(*F* ^2^)]		0.060
No. of reflections (individual, observed)	88 532, 59 317	
No. of parameters	2016	1734
H-atom treatment	Mixed	Constrained
Weighting scheme	*w* = 1/[σ^2^(*F*) + 0.0001*F* ^2^]	
Δρ_max_, Δρ_min_ (e Å^−3^)	1.64, −1.30	0.48, −0.21

**Table 3:** Intensity statistics of different reflection classes

Condition with respect to the supercell	*m*	〈*I*〉_all_	*n*_all_	〈*I*〉_obs_	*n*_obs_
4*h* + *k* = 8*n*	0	162	81 978	314	41 596
4*h* + *k* = 8*n* + 4 ± 1	±1	68	163 894	139	76 905
4*h* + *k* = 8*n* ± 2	±2	43	163 926	95	69 711
4*h* + *k* = 8*n* ± 1	±3	49	163 979	108	70 845
4*h* + *k* = 8*n* + 4	±4	31	82 033	73	31 209
