# Supplementary material for: On the puzzling case of sodium saccharinate 1.875-hydrate: structure description in (3+1)-dimensional superspace
Source: Acta Crystallogr B Struct Sci Cryst Eng Mater. 2020 Feb 1;76(Pt 1):18–27. doi: 10.1107/S2052520619014938 (PMC8788847; doi:10.1107/S2052520619014938)
Supplement: Supplementary file 4 [file b-76-00018-sup4.pdf]

STRUCTURAL SCIENCE
CRYSTAL ENGINEERING
MATERIALS

Volume 76 (2020)

Supporting information for article:

On the puzzling case of sodium saccharinate 1.875-hydrate: structure description in (3+1)-dimensional superspace

Toms Rekis, Andreas Schönleber and Sander van Smaalen

Supporting Information

On the Puzzling Case of Sodium Saccharinate 1.875-Hydrate: Structure Description in (3+1)-Dimensional Superspace

Toms Rekis, Andreas Schönleber, and Sander van Smaalen*

smash@uni-bayreuth.de

Laboratory of Crystallography, University of Bayreuth, 95440 Bayreuth, Germany

Table S1. Distances of the saccharinate anion atoms from the best planes through the corresponding phenyl rings (C1-C6). Distances given in Å. Molecule numbering (#1 – #16) corresponds to the superstructure (disordered molecule #13, B *t* = 0.625 omitted).

	**#1**	**#14**	**#3**	**#16**	**#5**	**#10**	**#7**	**#12**	**#2**	**#15**	**#4**	**#9**	**#6**	**#11**	**#8**
	**A<br>t=0.125**	**A<br>t=0.250**	**A<br>t=0.375**	**A<br>t=0.500**	**A<br>t=0.625**	**A<br>t=0.750**	**A<br>t=0.875**	**A<br>t=1.000**	**B<br>t=0.125**	**B<br>t=0.250**	**B<br>t=0.375**	**B<br>t=0.500**	**B<br>t=0.750**	**B<br>t=0.875**	**B<br>t=1.000**
C1	-0.0014	0.0044	0.0062	0.0061	0.0017	-0.0054	-0.0027	-0.0035	0.0029	0.0019	0.0124	-0.0005	0.0125	-0.0181	-0.0032
C2	0.0031	-0.0058	0.0000	-0.0061	-0.0009	-0.0001	0.0043	0.0001	-0.0015	-0.0036	-0.0093	-0.0017	-0.0123	0.0125	0.0075
C3	-0.0023	0.0026	-0.0068	0.0012	-0.0013	0.0057	-0.0018	0.0034	-0.0017	0.0025	-0.0023	0.0028	0.0034	0.0032	-0.0054
C4	-0.0010	0.0033	0.0067	0.0055	0.0029	-0.0055	-0.0037	-0.0037	0.0037	0.0016	0.0143	-0.0011	0.0088	-0.0157	-0.0035
C5	0.0032	-0.0052	0.0011	-0.0058	-0.0020	-0.0011	0.0061	-0.0001	-0.0022	-0.0041	-0.0098	-0.0018	-0.0105	0.0092	0.0090
C6	-0.0017	0.0006	-0.0075	-0.0011	-0.0005	0.0066	-0.0020	0.0041	-0.0015	0.0018	-0.0049	0.0025	-0.0030	0.0086	-0.0049
C7	0.0287	-0.0478	-0.0169	-0.0345	0.0178	0.0144	0.0517	-0.0050	-0.0203	-0.0472	-0.0456	-0.0222	-0.1088	0.0441	0.0730
N1	0.0586	-0.0806	0.0173	-0.0302	0.0507	-0.0533	0.1014	-0.0741	0.0049	-0.0756	-0.0036	-0.0610	-0.1039	0.0305	0.0907
S1	0.0168	-0.0326	0.0244	0.0148	0.0385	-0.0428	0.0264	-0.0369	0.0036	-0.0197	0.1250	0.0143	0.0392	-0.1284	0.0434
O1	1.2277	1.1961	1.2723	1.2456	1.2579	1.1640	1.2330	1.1753	1.1997	1.1835	1.4430	1.2717	1.3097	0.9634	1.2876
O2	-1.2053	-1.2334	-1.1727	-1.1919	-1.1710	-1.2662	-1.2029	-1.2613	-1.2322	-1.2498	-0.9745	-1.1317	-1.1244	-1.4531	-1.1435

Table S2. Conformer energies of 15 asymmetric saccharinate anions (disordered molecule #13, B *t* = 0.625 omitted). Gas phase energy used as the reference value.

Saccharinate	a.u.	kJ/mol
#1	-947.712903235	9.80
#2	-947.711998845	12.18
#3	-947.712052098	12.04
#4	-947.711882331	12.49
#5	-947.712322491	11.33
#6	-947.711384616	13.79
#7	-947.712241789	11.54
#8	-947.711681376	13.01
#9	-947.710933345	14.98
#10	-947.712833526	9.99
#11	-947.712015148	12.14
#12	-947.713051699	9.42
#14	-947.711776284	12.76
#15	-947.711935204	12.35
#16	-947.713272768	8.83
gas	-947.716637703	0.00

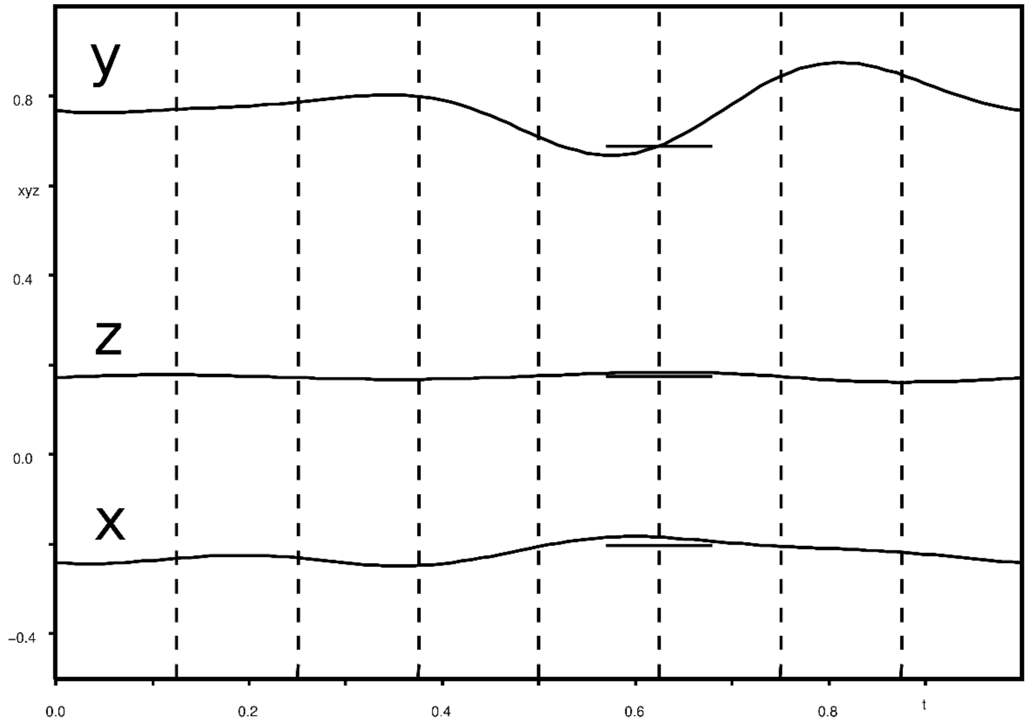

Fig. S1. Modulation functions of atom S1b (extended to cover full *t* range). Horizontal lines depict position of S1b' atom. Commensurate *t* sections depicted with dashed lines.

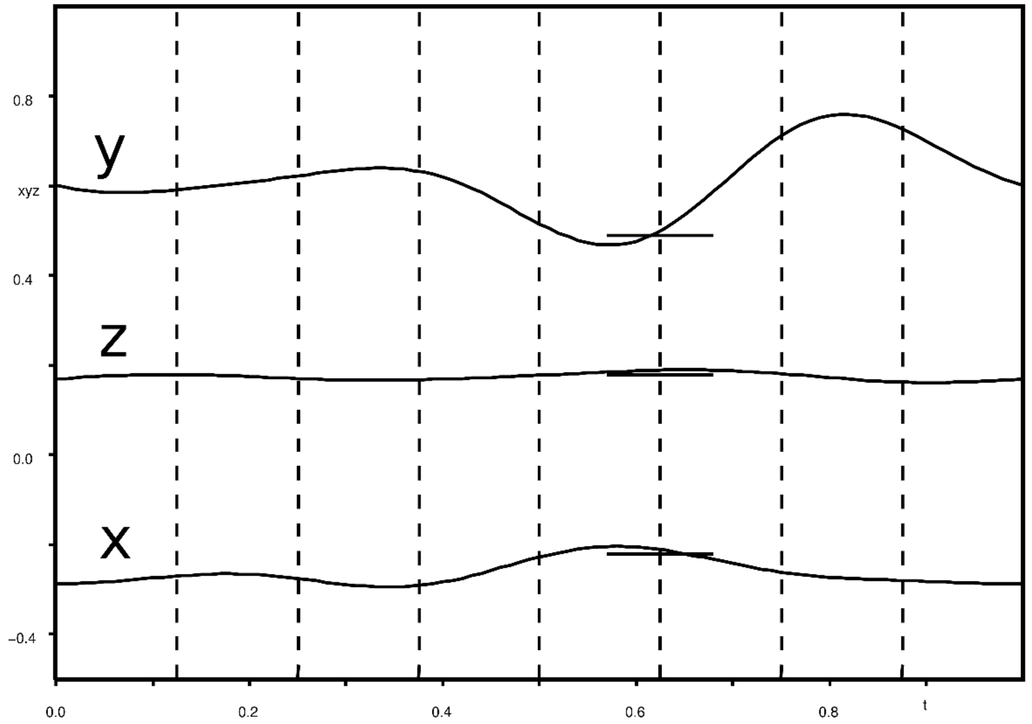

Fig. S2. Modulation functions of atom O1b (extended to cover full *t* range). Horizontal lines depict position of O1b' atom. Commensurate *t* sections depicted with dashed lines.

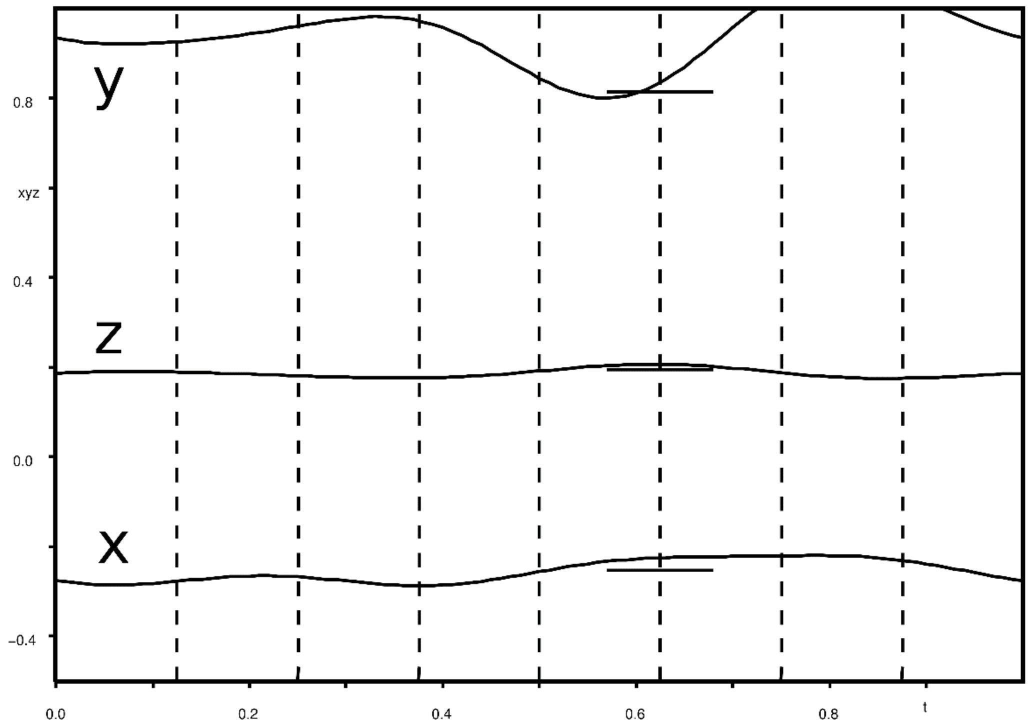

Fig. S3. Modulation functions of atom O2b (extended to cover full *t* range). Horizontal lines depict position of O2b' atom. Commensurate *t* sections depicted with dashed lines.

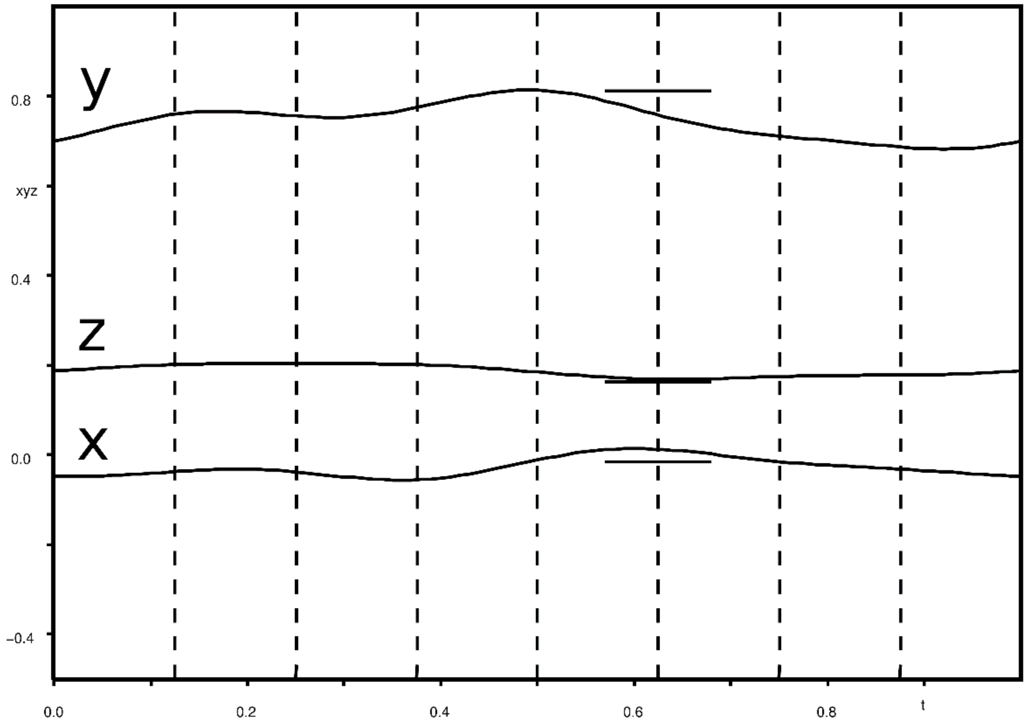

Fig. S4. Modulation functions of atom O3b (extended to cover full *t* range). Horizontal lines depict position of O3b' atom. Commensurate *t* sections depicted with dashed lines.

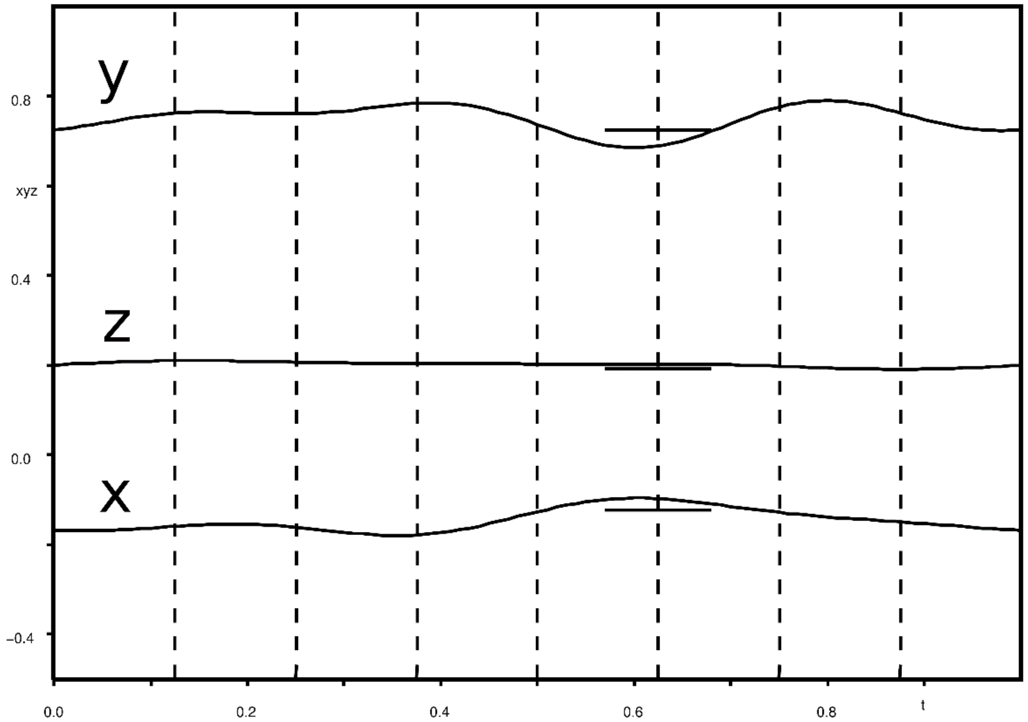

Fig. S5. Modulation functions of atom N1b (extended to cover full *t* range). Horizontal lines depict position of N1b’ atom. Commensurate *t* sections depicted with dashed lines.

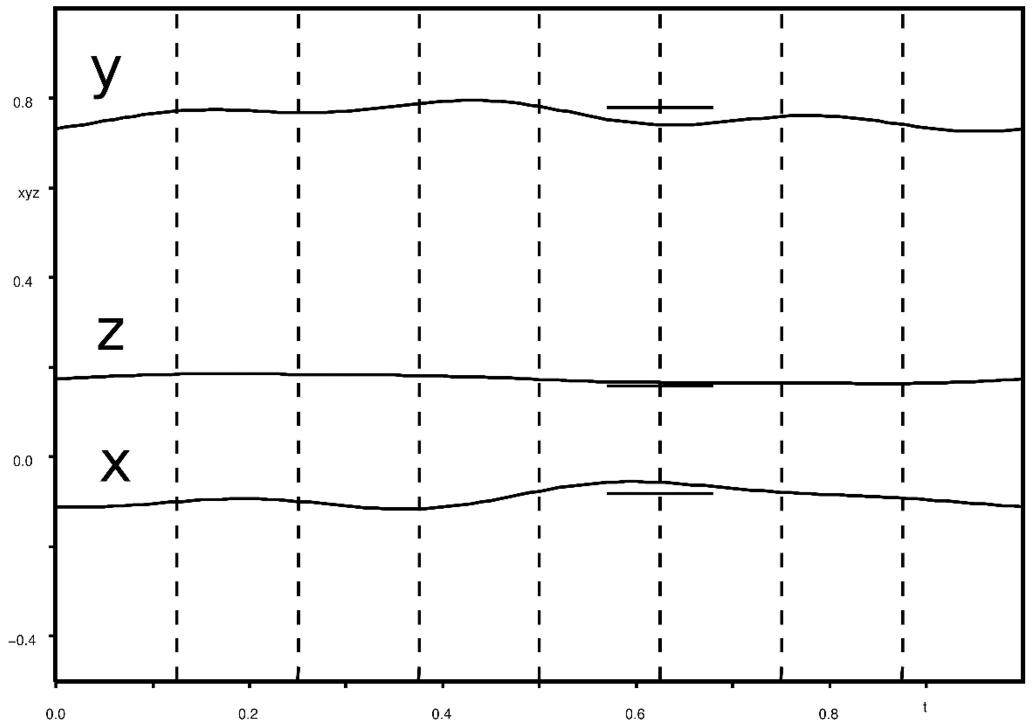

Fig. S6. Modulation functions of atom C7b (extended to cover full *t* range). Horizontal lines depict position of C7b' atom. Commensurate *t* sections depicted with dashed lines.
